# Supplementary material for: Effectiveness and cost-effectiveness of implementing HIV testing in primary care in East London: protocol for an interrupted time series analysis
Source: BMJ Open. 2017 Dec 14;7(12):e018163. doi: 10.1136/bmjopen-2017-018163 (PMC5735409; doi:10.1136/bmjopen-2017-018163)
Supplement: Author's manuscript [file bmjopen-2017-018163.draft_revisions.pdf]

BMJ Open is committed to open peer review. As part of this commitment we make the peer review history of every article we publish publicly available.

When an article is published we post the peer reviewers' comments and the authors' responses online. We also post the versions of the paper that were used during peer review. These are the versions that the peer review comments apply to.

The versions of the paper that follow are the versions that were submitted during the peer review process. They are not the versions of record or the final published versions. They should not be cited or distributed as the published version of this manuscript.

BMJ Open is an open access journal and the full, final, typeset and author-corrected version of record of the manuscript is available on our site with no access controls, subscription charges or pay-per-view fees (http://bmjopen.bmj.com).

If you have any questions on BMJ Open's open peer review process please email editorial.bmjopen@bmj.com

BMJ Open

Effectiveness and cost effectiveness of implementing HIV testing in primary care in east London: protocol for an interrupted time series analysis

Journal:	*BMJ Open*
Manuscript ID	bmjopen-2017-018163
Article Type:	Protocol
Date Submitted by the Author:	16-Jun-2017
Complete List of Authors:	Leber, Werner; Queen Mary University of London, Centre for Primary Care and Public Health<br>Beresford, Lee; Queen Mary University of London<br>Nightingale, C; St George's University London, Division of Population Health Sciences and Education<br>Capelas Barbosa, Estela; University College London<br>Morris, Stephen; University College London, Department of Applied Health Research<br>El-Shogri, Farah; Queen Mary University of London<br>McMullen, Heather; Queen Mary University of London, Blizard Institute<br>Boomla, Kambiz; Barts and The London School of Medicine and Dentistry, Queen Mary University of London, Centre for Primary Care and Public Health<br>Delpech, Valerie; Public Health England<br>Brown, Alison; Public Health England<br>Hutchinson, Jane; Barts Health NHS Trust<br>Apea, Vanessa; Barts Health NHS Trust<br>Symonds, Merle; Barts Health NHS Trust<br>Gilliham, Smantha; Barts Health NHS Trust<br>Creighton, S.; Homerton Univ Hosp<br>Shahmanesh, Maryan; University College London<br>Fulop, Naomi; University College London, Applied Health Research<br>Estcourt, Claudia; Glasgow Caledonian University; Barts Health NHS Trust<br>Anderson, Jane ; Homerton University Hospital, Centre for the study of Sexual Health and HIV<br>Figueroa, Jose; NHS England<br>Griffiths, Chris; Barts and The London School of Medicine and Dentistry,
**Primary Subject Heading**:	General practice / Family practice
Secondary Subject Heading:	HIV/AIDS, Public health
Keywords:	PRIMARY CARE, HIV & AIDS < INFECTIOUS DISEASES, Public health < INFECTIOUS DISEASES

For peer review only

Effectiveness and cost effectiveness of implementing HIV testing in primary care in east London: protocol for an interrupted time series analysis

Werner Leber[1]
Lee Beresford[1]
Claire Nightingale[2]
Estela Capelas Barbosa[3]
Steve Morris[3]
Farah El-Shogri[1]
Heather McMullen[1]
Kambiz Boomla[1]
Valerie Delpech[4]
Alison Brown[4]
Jane Hutchinson[5]
Vanessa Apea[5]
Merle Symonds[5]
Samantha Gilliham[5]
Sarah Creighton[6]
Maryam Shahmanesh[3]
Naomi Fulop[3]
Claudia Estcourt[5,7]
Jane Anderson[6]
Jose Figueroa[8]
Chris Griffiths[1]

1. Centre for Primary Care and Public Health, Queen Mary University of London
2. Population Health Research Institute, St George's, University of London
3. Department of Applied Health Research, University College London
4. Public Health England
5. Barts Health NHS Trust
6. Centre for Sexual Health, Homerton University Hospital NHS Foundation Trust

For peer review only
For peer review only - http://bmjopen.bmj.com/site/about/guidelines.xhtml

7. Glasgow Caledonian University
8. NHS England

Correspondence to:
Werner Leber
Centre for Primary Care and Public Health
Barts and The London School of Medicine & Dentistry
Queen Mary University of London, London E1 2AB
w.leber@qmul.ac.uk

Sponsor: Mr Gerry Leonard, Queen Mary University of London, gerry.leonard@bartshealth.nhs.uk

Key words: HIV; HIV testing; HIV screening; HIV diagnosis; implementation; interrupted time series; cost-effectiveness

Protocol version: v5

Date: 14 June, 2017

Abstract

Introduction

HIV remains underdiagnosed. Guidelines recommend routine HIV testing in primary care but evidence on implementing testing is lacking. In the RHIVA2 cluster randomised controlled trial (RCT), we showed that providing training and rapid point-of-care HIV screening at general practice registration in Hackney led to cost-effective increased and earlier diagnosis of HIV. However, interventions effective in a trial context may be less so when implemented in routine practice. We describe the protocol for an MRC phase IV implementation programme, evaluating the impact of the post-RCT implementation of the RHIVA2 intervention into routine care in Hackney. We will examine if the RHIVA2 post-trial implementation programme is a) effective and cost-effective (longitudinal study) and b) comparable to a long-standing general practice-improved service promoting HIV testing in a neighbouring borough, but superior to usual care provided in another adjacent borough (cross-sectional study).

For peer review only - http://bmjopen.bmj.com/site/about/guidelines.xhtml

Methods and analysis

Service evaluation using interrupted time series and cost-effectiveness analyses. We will include all general practices in three contiguous high HIV prevalence east London boroughs; Hackney, Tower Hamlets, and Newham. All adults aged 16 and above registered with the practices will be included. The interventions to be examined are, in Hackney: the RHIVA2 RCT and a post-trial implementation programme (including practice-based education and training, an external quality assessment, incentive payments for rapid HIV testing, and incorporation of rapid HIV testing in the sexual health Local Enhanced Service); in Tower Hamlets: the general practice sexual health Network Improved Service; and in Newham: usual care. Co-primary outcomes are rates of HIV testing and new HIV diagnoses.

Ethics and dissemination

The chair of the Camden & Islington NHS Research Ethics Committee London has endorsed this programme as an evaluation of routine care. Study results will be published in peer-reviewed journals and reported to commissioners.

Strengths and limitations of this study:

- We will use existing trial data preceding the implementation programme in addition to post-trial data which will be collected using similar methods to those used in the trial.
- We will remotely extract fully anonymised general practice data across all participating boroughs using a single general practice computer system (Egton Medical Information Systems, EMIS).
- We expect that the large dataset with at least 12 time points before and at least 27 time points after the implementation will provide precise estimation of the effects of intervention using interrupted time series modelling.
- Differences in implementation protocols between Hackney and Tower Hamlets, and missing data, such as rapid HIV testing pilots in a small number of Newham practices, may affect comparability between boroughs.

For peer review only - http://bmjopen.bmj.com/site/about/guidelines.xhtml

Introduction

HIV prevalence is increasing, with over 100,000 people now estimated to be living with HIV in the UK.[1] From 1999 to 2013, the numbers of patients accessing specialist care increased four-fold.[2 3] Successfully treated, HIV now has characteristics of a long-term condition, with patients taking lifelong treatment; furthermore it increasingly exemplifies the clustering of multiple morbidities, both with other infectious (e.g. tuberculosis, viral hepatitis, and human papilloma virus) and other long term conditions (e.g. cardiovascular disease, osteoporosis and mental illness). The cost of HIV care for the NHS (without considering the cost of treating co-morbidities) in 2013 was estimated to be £750m (£1bn with social care included).[4]

Late diagnosis of HIV increases morbidity and mortality, and is associated with unwitting onward transmission. To reduce the pool of undiagnosed infection in the population, the British HIV Association (BHIVA, 2008) and the National Centre for Health and Care Excellence (NICE, 2011, 2016) recommended expansion of HIV testing from sexual health and antenatal clinics to non-traditional settings including general practices located in high HIV prevalence areas (2 or more people diagnosed with HIV/1000 adult population).[5-8] Although National Policy for HIV screening in primary care is lacking, it was hoped that recommendation of routine HIV testing would enhance awareness of HIV amongst GPs and become standard practice in primary care.[9] Pilot studies have demonstrated feasibility and acceptability of routine HIV testing amongst patients and health care professionals.[10 11] Despite this however a recent systematic review identified low uptake of routine HIV testing in non-traditional settings including general practice following the BHIVA 2008 guidelines.[12]

The RHIVA2 implementation programme

We conducted a large-scale pragmatic cluster randomised controlled trial (RHIVA2) across a single London borough showing that rapid HIV testing offered at general practice registration was effective and safe, and delivered increased and earlier HIV diagnosis.[13] In addition, a recent health economics modelling analysis of RHIVA2 demonstrated that HIV screening in high prevalence primary care is cost-effective (Baggaley et al; accepted for publication by The Lancet HIV). Following completion of the RHIVA2 trial, we implemented HIV testing into routine care across all Hackney practices using a theory-based programme[14-16] comprising an adaptation of

For peer review only

the original RHIVA2 practice-based education programme, a borough-wide evaluation of HIV care, and introduction of payments for HIV testing as part of an updated general practice sexual health Local Enhanced Service (LES).

We evaluated the RHIVA2 trial and implementation programme by designing an MRC phase IV implementation study.[17] The study also compares the impact of its implementation with that of a general practice sexual health Network Improved Service (NIS) promoting HIV testing in the neighbouring borough of Tower Hamlets, and with usual care in the neighbouring borough of Newham.

Figure 1 summarises the timelines of HIV-related interventions in these three boroughs.

The study will therefore address the following questions:

- How effective and cost-effective is the RHIVA2 intervention when implemented as a routine clinical service outside a research context?
- How do effectiveness and cost-effectiveness of the RHIVA2 trial and implementation programme compare with introduction of a routine borough-wide sexual health NIS promoting routine HIV testing in Tower Hamlets?
- How do both the RHIVA2 trial and post-trial implementation programme and the Tower Hamlets sexual health NIS compare to Newham that offers usual HIV care only?

Methods and analysis

Setting

The study will be set in the three east London boroughs of Hackney, Tower Hamlets, and Newham. The estimated diagnosed HIV prevalence for these boroughs is 8.1, 6.6, and 6.9 per 1000 adult population respectively. We will include all adults aged 16 and above registered with general practices in the three boroughs.

For peer review only - http://bmjopen.bmj.com/site/about/guidelines.xhtml

Design

Service evaluation comprising a pragmatic cohort using an interrupted time series (ITS) analysis and cost effectiveness analysis. The evaluation will examine the longitudinal impact of interventions in Hackney and the comparative cross-sectional impact of interventions in Hackney, Tower Hamlets, and Newham. HIV testing and diagnosis data will be collected from April 01, 2009 to June 30, 2015.

Interventions

Hackney

The RHIVA2 trial programme was introduced in April 2010 in 20 general practices randomized to the intervention arm of the RHIVA2 cluster randomized trial (Table 1). This theory-based intervention is described in detail in Leber et al. 2015,[13] and consisted of a practice-based educational training session for the primary care team to promote rapid HIV testing at registration, a follow up meeting with a nominated practice lead nurse, and an incentive payment of £10 per rapid HIV test performed. Testing competence was monitored by an external quality assessment.

The RHIVA2 post-trial implementation programme ran from September 2012 to April 2015 and comprised the following three theory-based components:[14-16]

1) Post-trial HIV testing training (September 2012). This was offered to all Hackney practices at the completion of the RHIVA2 trial and comprised a modification of the original RHIVA2 practice based education session whereby practices were encouraged to offer both serology and rapid HIV testing in any clinical setting.
2) HIV care service evaluation (October 2012 to March 2013) of missed diagnoses of HIV and safety of co-prescribing with anti-retroviral medication in Hackney general practice.[18]
3) Hackney sexual health LES. From April 2013, the existing LES (incentive payments of £265 per patient newly diagnosed) were replaced with HIV testing payments to practices of between £7 and £10 for every rapid or serology HIV test carried out in any clinical setting, in addition to a payment £258 for any new diagnosis.

For peer review only - http://bmjopen.bmj.com/site/about/guidelines.xhtml

Tower Hamlets

A sexual health NIS promoting HIV testing was introduced In April 2011, replacing the previous sexual health Local Enhanced Service (incentive payments of £25 for sexual health screening including offer of HIV test initially), and included payments of £10 per HIV test (combined with or without hepatitis B and syphilis testing) for patients attending the new patient check and sexual health consultations. A primary care facilitator in sexual health (JH) provides regular support and performance feedback to the practices.

Newham

Usual care – no specific initiatives were promoted relating to HIV testing in primary care.

The RHIVA2 intervention is based on published clinician behaviour change programmes,[14-16] and the Tower Hamlets NIS follows Royal College of General Practitioners guidance on establishing general practice federations.[19] We will report our study in accordance with the STARI reporting guidelines for implementation studies.[20]

Outcome measures

All data gathered will comprise routine clinical data. No identifiable data will be held by the research team.

Co-primary outcome measures

Co-primary outcome measures will be:

- Rates of HIV testing (combined rapid and serology testing; number of patients tested/1000 population per year)
- Rates of new HIV diagnosis (number of newly diagnosed patients/10,000/year)

For peer review only - http://bmjopen.bmj.com/site/about/guidelines.xhtml

Secondary outcome measures

Secondary outcome measures will include data on type of HIV test (serology; rapid), location of diagnosis, stage of disease and linkage to secondary care and retention in secondary care:

- Number / rate of patients who received a rapid HIV test
- Number / rate of patients who received a serology HIV test
- Number / rate of patients newly diagnosed in general practice
- Mean and median CD4 count of patients newly diagnosed in primary care
- Proportion of patients with a CD4 count <350 cells per μL blood
- Proportion of patients with a CD4 count <200 μL blood.
- The proportion of patients newly diagnosed with HIV in primary care that attend an HIV specialist department within 2 weeks of diagnosis (BHIVA Standards of Care for PLHIV, Standard 2, 2013).[21]
- The proportion of patients newly diagnosed with HIV in primary care that attend an HIV specialist department within 4 weeks of diagnosis (NHS England Standard)
- The proportion of patients newly diagnosed with HIV in primary care that attend a HIV specialist department within 3 months of diagnosis (Optimising testing and linkage to care for HIV across Europe, OptTEST, 2013)
- Number of patients previously known to have HIV, who attend an HIV specialist clinic between 12 and 24 months following new diagnosis in primary care (BHIVA Standards of Care for PLHIV, Standard 2, 2013).

Data sources

We will gather data from April 2009 to June 2015 using the following sources:

Organisational data:

For Hackney practices, we hold detailed records of RHIVA2 trial educational training sessions, post-trial education sessions, and participation in the borough-wide service evaluation. For Tower Hamlets, we hold data on timing of the introduction of the NIS. For all boroughs, we will seek to obtain any other relevant activities promoting HIV testing in the practices.

For peer review only - http://bmjopen.bmj.com/site/about/guidelines.xhtml

Clinical data:

HIV diagnosis data

Borough-specific master case-report forms (CRF) containing lists of patients with a positive confirmatory HIV test result will be generated by the lead HIV clinician from the clinical HIV services in each borough. This master CRF will be shared with the study team in an anonymised format as per the RHIVA2 protocol. At Homerton Hospital, Hackney data on newly diagnosed patients will be collected prospectively as outlined in RHIVA2 (continuous data available from 2009). For Tower Hamlets and Newham, new diagnosis data will be collected retrospectively for the same period. Clinical leads will liaise with Public Health England to categorise patients with a positive confirmatory HIV test into newly diagnosed patients and people already known to be HIV positive, and obtain data on patients who had defaulted from care between 12 and 24 months following diagnosis.

HIV testing data

In collaboration with the Clinical Effectiveness Group in the Centre for Primary Care and Public Health (QMUL), we will collect anonymised data on HIV testing in primary care, as per RHIVA2 protocol, using remote searches of borough-specific READ codes used for rapid and serology HIV testing.

Sample size and analysis

Sample size

Sample size calculations for the interrupted time series (ITS) analysis are based on the number of time points available to perform the analysis. Generally, a minimum of 10-12 time points before and after an intervention are required to determine statistical significance of an intervention.[22 23] 12 monthly time points are available before the RHIVA2 intervention, and a minimum of 34 time points for the period thereafter. For Tower Hamlets, 21 monthly time points are available before the introduction of the sexual health NIS, a minimum of 27 time points thereafter.

For peer review only - http://bmjopen.bmj.com/site/about/guidelines.xhtml

Longitudinal analysis of effectiveness of RHIVA2 implementation programme in Hackney

We will compare testing rates using an interrupted time series regression approach with a mixed Poisson model (or negative binomial model if there is overdispersion) to analyse the effect of interventions. Mixed Poisson regression will allow for the clustered nature of the data, i.e. allowing each practice to have a different intercept (baseline HIV testing rate). We will also adjust for practice list size as an offset variable in the analysis. Using this approach, it will be possible to examine whether there is a change in level and/or a change in regression slope following the implementation of the intervention.

We will firstly examine the impact of the RHIVA2 intervention on combined serology and rapid HIV testing rate in practices that received the RHIVA2 implementation (post-trial setting, comparing testing rates pre- and post-intervention). We will repeat this analysis including practices which did not receive the RHIVA2 intervention during the trial or the further implementation phase, to act as a comparator. This will allow comparison of testing rates in practices post-implementation to their pre-implementation rates, also adjusting for continuing trends in practices that did not receive the RHIVA2 implementation. We will also repeat the analysis using Newham practices (which received no intervention) as a further comparator.

The date of intervention will be defined as the date of the first RHIVA2 training session. We will conduct similar analysis to the above, with the endpoint of HIV diagnosis, rather than testing.

To assess sustainability, we will model testing rates in practices which received the RHIVA2 intervention during the trial period, considering an interaction term between time post-intervention and whether practices were retrained. This will allow an assessment to be made about whether retraining influences the slope post-intervention i.e. the sustainability of the RHIVA2 intervention.

Secondary analyses will explore differential impact of the introduction of an updated EMIS template that reflected the sexual health NIS specifications.

For peer review only - http://bmjopen.bmj.com/site/about/guidelines.xhtml

Cross-sectional analysis of effectiveness of the RHIVA2 implementation programme in Hackney, a sexual health NIS in Tower Hamlets, and usual care in Newham

We will examine the impact of the sexual health NIS on combined serology and rapid HIV testing rate in all Tower Hamlets practices (comparing testing rates pre- and post-implementation). We will repeat this analysis but also including practices in Newham, which received no intervention at any stage, to act as a comparator. The date of intervention will be defined as the date when the NIS system went live (April 2011). We will also compare RHIVA2 implementation in Hackney to the introduction of the sexual health NIS in Tower Hamlets.

We will conduct similar analysis to the above, with the endpoint of HIV diagnosis, rather than testing.

Cost-effectiveness analysis

Cost-effectiveness analyses for the longitudinal and cross-sectional analyses described above will build on a cost-effectiveness model developed for the RHIVA2 trial (manuscript in preparation). To assess value for money, we will identify the difference in costs and the difference in benefits of implementing the RHIVA2 programme and we aim to distinguish between two different analyses. First, we will evaluate the cost-effectiveness of the RHIVA2 using the same 'comparator' defined in the longitudinal analysis, i.e. practices which did not receive the RHIVA2 intervention at any stage will be considered the 'absence of the intervention'.

Second, we will build a cost-effectiveness comparison between different forms of HIV screening in East London, formally comparing the RHIVA2 implementation programme (conducted in Hackney) with the NIS (Tower Hamlets) and usual care (Newham). Once again, all three different approaches will be compared to practices, which did not receive the RHIVA2 intervention at any stage, as these are considered the 'absence of the intervention'. Given that the same control group of practices in Hackney will serve as reference, this second analysis will formally compare the different approaches from different boroughs, considering no intervention in Hackney as a reference.

For peer review only - http://bmjopen.bmj.com/site/about/guidelines.xhtml

Three main cost categories will be explored: (1) the extra start-up cost of RHIVA2 implementation (specifically the cost for RHIVA2 staff time, including follow up activities; cost of training; cost of training materials; and cost of incentives to practices); (2) the extra recurrent cost for performing rapid HIV testing; and (3) additional costs associated with reactive rapid HIV test results.

Benefits of the RHIVA2 programme will include health outcomes for patients, specifically deaths averted, secondary HIV infections averted and quality adjusted life years (QALYs); these are a function of the uptake of screening and screening positivity, which will be quantified directly by the analyses.

This study is a service evaluation which does not require registration as a trial.

Ethical aspects

The programme described is an evaluation of routine care. Its status as such has been endorsed by the chair of the Camden & Islington NHS Research Ethics Committee London. The Committee's view is that the work does not require NHS ethics approval. The programme will be implemented in partnership with the local providers and commissioners and involve the analysis of existing routine data. This also applies to data on delayed diagnosis of HIV (CD4 count), and demographics reported in RHIVA2 trial, which is routinely collected as part of a national audit (BHIVA National Audit 2010) and has been included in Public Health Outcomes Framework (http://www.phoutcomes.info). We will inform the REC, the CCG, and Public Health about any important changes to the protocol.

This service evaluation is to answer the question: what standard does this service achieve? It involves routine implementation of an intervention outside of the research context. The actual management options are those of the clinician and patient only. There is no random allocation to intervention and control groups. No individual consent is required from newly diagnosed patients.

For peer review only - http://bmjopen.bmj.com/site/about/guidelines.xhtml

In addition to CD4 count and viral load the following additional data will be obtained by the clinical leads of specialist departments in an anonymized fashion and stored in accordance with good clinical practice:

- Most likely mode of HIV acquisition
- Sexual orientation
- Soundex code
- Ethnicity
- Gender
- Age at diagnosis

Discussion

The MRC Framework for Complex Interventions[24] clearly describes the need for implementation studies that test the effectiveness of interventions found to be promising in Phase III randomized controlled trials. Successful translation of such interventions into routine care cannot be assumed; trials take place in tightly controlled conditions that aim to limit bias, with selected (often motivated) participants, whilst implementation programmes (usually) aim much more broadly to include all potential participants.[25] Trial interventions will often need to be augmented by a distinct implementation package. Their success will be affected by multiple factors including, but not limited to contextual factors such as, differences in setting, population, organization of health care systems, competing initiatives, and financial aspects (including availability of incentives). Evaluations of implementation of trials into routine care are frequently not carried out, and if they are, are often poorly reported.[26] Such evaluations are highly unlikely to involve randomised designs, and are more likely to be observational, including the use of service evaluations. Designs are often constrained by health systems and data availability, but need to be as robust as possible. New reporting guidelines have recently been published that highlight the need for evaluation of implementation and aim to improve their reporting, such that they can be more easily accessed in the literature and serve to guide others in designing the most effective implementation programmes.[25]

For peer review only - http://bmjopen.bmj.com/site/about/guidelines.xhtml

Recent NICE Guidelines recommend expanded HIV testing in primary care,[6 7 27] but little information is available about how to operationalize testing, especially in the context of current resource constraints in primary care. It is therefore vital that innovative and effective ways are found to implement testing. The strengths of the proposed work include the availability of existing trial data preceding the implementation programme, the capacity to collect data longitudinally using similar methods to those used in the trial, and the use by general practices across all three participating boroughs of a single GP computer system supplier (EMIS), data from which can be remotely and electronically (with full anonymisation) searched and collated by the research team (http://www.blizard.qmul.ac.uk/ceg-home.html), confirmation of HIV test results for all study practices at the same tertiary care laboratory (Barts Health NHS Trust), and external data validation by Public Health England. This approach provides a unique capability, allowing comparative data on three separate approaches to HIV testing and diagnosis to be accurately gathered and analysed.

The results of the proposed analysis are likely to be of considerable interest to commissioners, particularly given that late diagnosis of HIV is a UK Public Health Outcomes Framework priority,[28] and that cost effectiveness of HIV testing is complex, and becoming cost-effective in trial-based models only after a period of time has elapsed to counter the additional significant costs of early treatment.

Study status

We have completed collection of HIV testing data for all three boroughs. The HIV diagnosis and cost-effectiveness data for this study are expected to become available in the next six to nine months.

Abbreviations

BHIVA, British HIV Association

CLAHRC, Collaboration for Leadership in Applied Health Research and Care

EMIS, Egton Medical Information Systems

For peer review only - http://bmjopen.bmj.com/site/about/guidelines.xhtml

HIV, Human Immunodeficiency Virus

MRC, Medical Research Council

NICE, The National Institute for Health and Care Excellence

NIHR, National Institute for Health Research

LES, Local Enhanced Service

NIS, Network Improved Service

RCT, Randomised controlled trial

RHIVA2, Rapid HIV Testing Assessment 2

Competing interests

JA reports fees and non-financial support from Bristol Myers Squibb, grants and personal fees from Gilead Sciences, personal fees from ViiV, personal fees from Merck Sharp & Dohme, grants from Janssen, and personal fees from AbbVie, outside the submitted work. The remaining authors and contributors declare no competing interest.

Author's contributions

WL, LB, CN, ECB, SM, CE, JA, JF, and CG significantly contributed to designing the study and drafted the protocol. FES, HM, KB, VD, AB, JH, AV, MS, SG, SC, MS, and NF contributed to designing the study. All authors and contributors approved the submitted version of the manuscript.

Funding

WL, FS, LB, ECB were supported by the National Institute for Health Research (NIHR) Collaboration for Leadership in Applied Health Research and Care (CLAHRC) North Thames at Barts Health NHS Trust. The views expressed are those of the author(s) and not necessarily those of the NHS, the NIHR or the Department of Health. HM was supported by a National Institute of Health Research Doctoral Fellowship from 2013 to 2016.

For peer review only - http://bmjopen.bmj.com/site/about/guidelines.xhtml

Role of the funding source

JF, a clinician employed by NHS City and Hackney, which funded the study, was involved in designing the study, data interpretation, and writing the report, but has no role in data collection or analysis. Neither the Department of Health nor the study sponsor had any role in any aspect of the study. The corresponding author will have full access to all the data in the study and has final responsibility for the decision to submit for publication.

Roles and responsibilities

CG and WL are the co-chief investigators of the study and formed the steering committee with SM, CE, JH, MS, JA, and NF. WL is responsible for the data management. CN and LB have produced the power calculation and statistical model. ECB and SM have developed the protocol for the health economics analysis.

Data Sharing Statement

This study is an evaluation of service, and no ethical approval is available for data sharing.

References

1. Public Health England. HIV in the UK: 2016 report, 2016.
2. Health Protection Agency. HIV in the United Kingdom: 2009 Report, 2009.
3. Public Health England. HIV in the United Kingdom: 2014 report, 2014.
4. Mandalia S, Mandalia R, Lo G, et al. Rising population cost for treating people living with HIV in the UK, 1997-2013. 2010;5(12):e15677.
5. British HIV Association. UK National Guidelines for HIV testing, 2008.
6. National Institute for Health and Care Excellence. Increasing the uptake of HIV testing to reduce undiagnosed infection and prevent transmission among men who have sex with men, 2011.
7. National Institute for Health and Care Excellence. Increasing the uptake of HIV testing to reduce undiagnosed infection and prevent transmission among black African communities living in England, 2011.
8. National Institute for Health and Care Excellence. HIV testing: increasing uptake among people who may have undiagnosed HIV, 2016.

For peer review only - http://bmjopen.bmj.com/site/about/guidelines.xhtml

9. Kall MM, Smith RD, Delpech VC. Late HIV diagnosis in Europe: a call for increased testing and awareness among general practitioners. *Eur J Gen Pract* 2012;18(3):181-6. doi: 10.3109/13814788.2012.685069
10. Health Protection Agency. Time to Test for HIV: Expanding HIV testing in healthcare and community services in England, 2011.
11. Prost A, Elford J, Imrie J, et al. Social, behavioural, and intervention research among people of Sub-Saharan African origin living with HIV in the UK and Europe: literature review and recommendations for intervention. *AIDS and behavior* 2008;12(2):170-94. doi: 10.1007/s10461-007-9237-4
12. Elmahdi R, Gerver SM, Gomez Guillen G, et al. Low levels of HIV test coverage in clinical settings in the U.K.: a systematic review of adherence to 2008 guidelines. *Sex Transm Infect* 2014;90(2):119-24. doi: 10.1136/sextrans-2013-051312
13. Leber W, McMullen H, Anderson J, et al. Promotion of rapid testing for HIV in primary care (RHIVA2): a cluster-randomised controlled trial. *The Lancet HIV* 2015;2(6):e229-e35. doi: 10.1016/S2352-3018(15)00059-4
14. Forsetlund L, Bjorndal A, Rashidian A, et al. Continuing education meetings and workshops: effects on professional practice and health care outcomes. *The Cochrane database of systematic reviews* 2009(2):CD003030. doi: 10.1002/14651858.CD003030.pub2
15. Griffiths C, Sturdy P, Brewin P, et al. Educational outreach to promote screening for tuberculosis in primary care: a cluster randomised controlled trial. *Lancet* 2007;369(9572):1528-34. doi: 10.1016/s0140-6736(07)60707-7 [published Online First: 2007/05/08]
16. O'Brien MA, Rogers S, Jamtvedt G, et al. Educational outreach visits: effects on professional practice and health care outcomes. *The Cochrane database of systematic reviews* 2007(4):CD000409. doi: 10.1002/14651858.CD000409.pub2
17. Campbell M, Fitzpatrick R, Haines A, et al. Framework for design and evaluation of complex interventions to improve health. *BMJ* 2000;321(7262):694-6.
18. Wellesley R, Whittle A, Figueroa J, et al. Does general practice deliver safe primary care to people living with HIV? A case-notes review. *Br J Gen Pract* 2015;65(639):e655-61. doi: 10.3399/bjgp15X686905 [published Online First: 2015/09/29]
19. Royal College of General Practitioners. The 2022 GP. A Vision for General Practice in the future NHS, 2014.
20. Pinnock H, Epiphaniou E, Sheikh A, et al. Developing standards for reporting implementation studies of complex interventions (StaRI): a systematic review and e-Delphi. *Implementation Science* 2015;10(1):42.
21. British HIV Association. Standards of care for people living with HIV in 2013, 2013.
22. Ramsay CR, Matowe L, Grilli R, et al. Interrupted time series designs in health technology assessment: lessons from two systematic reviews of behavior change strategies. *Int J Technol Assess Health Care* 2003;19(4):613-23.
23. Wagner AK, Soumerai SB, Zhang F, et al. Segmented regression analysis of interrupted time series studies in medication use research. *J Clin Pharm Ther* 2002;27(4):299-309. [published Online First: 2002/08/14]
24. Campbell NC, Murray E, Darbyshire J, et al. Designing and evaluating complex interventions to improve health care. *BMJ* 2007;334(7591):455-9. doi: 10.1136/bmj.39108.379965.BE
25. Pinnock H, Epiphaniou E, Sheikh A, et al. Developing standards for reporting implementation studies of complex interventions (StaRI): a systematic review and e-Delphi. *Implement Sci* 2015;10(1):42. doi: 10.1186/s13012-015-0235-z

For peer review only - http://bmjopen.bmj.com/site/about/guidelines.xhtml

26. Newhouse R, Bobay K, Dykes PC, et al. Methodology issues in implementation science. *Medical care* 2013;51(4 Suppl 2):S32-40. doi: 10.1097/MLR.0b013e31827feeca
27. National institute for Health and Care Excellence. HIV testing: increasing uptake among people who may have undiagnosed HIV (Joint NICE and Public Health England guideline), 2016.
28. Public Health England. Public Health Outcomes Framework: February 2015 data update, 2015.

For peer review only

For peer review only - http://bmjopen.bmj.com/site/about/guidelines.xhtml

For peer review only

For peer review only - http://bmjopen.bmj.com/site/about/guidelines.xhtml

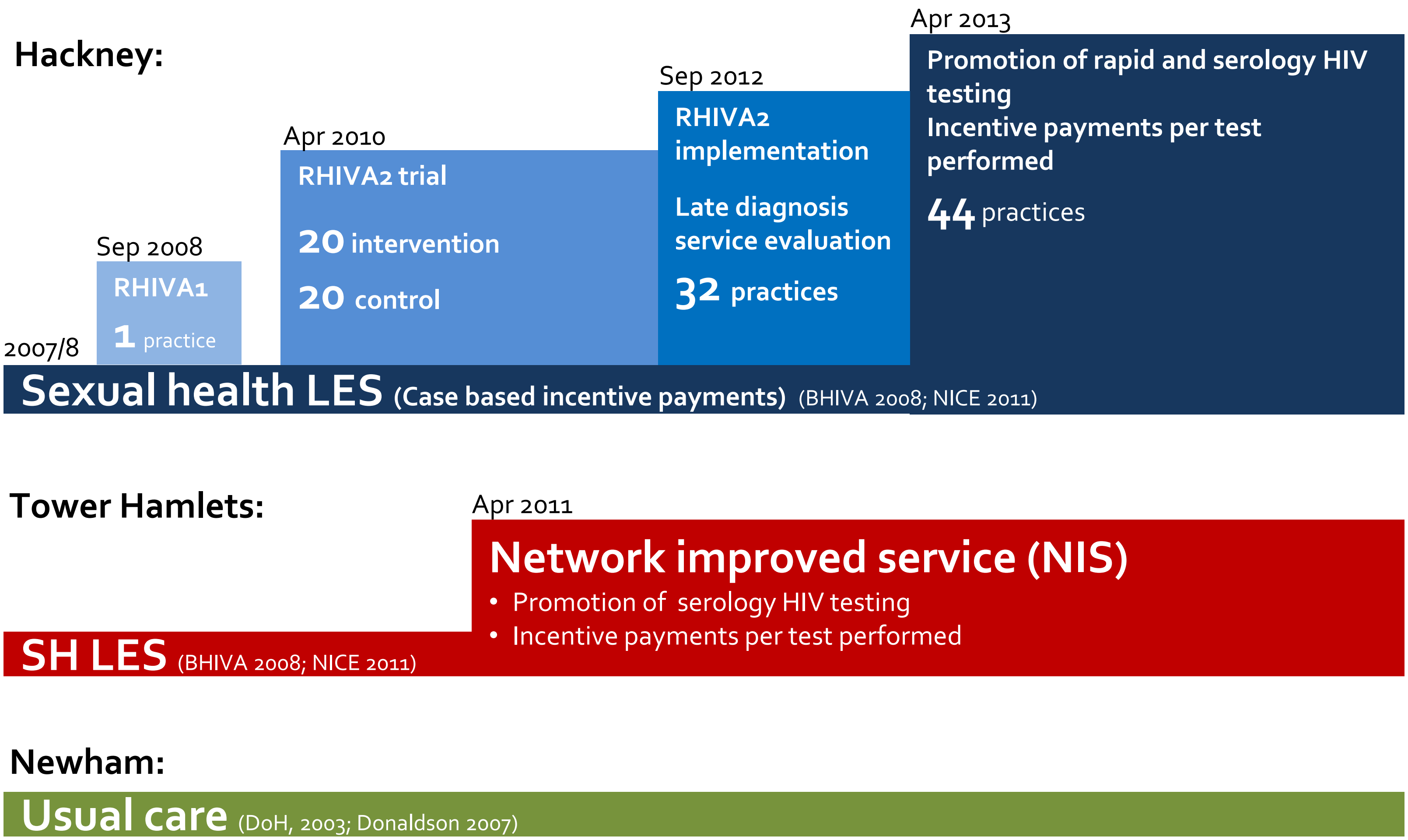
Hackney:
2007/8
Sep 2008
RHIVA1
1 practice
Apr 2010
RHIVA2 trial
20 intervention
20 control
Sep 2012
RHIVA2 implementation
Late diagnosis service evaluation
32 practices
Apr 2013
Promotion of rapid and serology HIV testing
Incentive payments per test performed
44 practices
Sexual health LES (Case based incentive payments) (BHIVA 2008; NICE 2011)
Tower Hamlets:
Apr 2011
Network improved service (NIS)
• Promotion of serology HIV testing
• Incentive payments per test performed
SH LES (BHIVA 2008; NICE 2011)
Newham:
Usual care (DoH, 2003; Donaldson 2007)

	STUDY PERIOD									
	Control period			Practice Allocation	Post-allocation			Close-out		
**TIMEPOINT**	**-48 months**	**-41 months**	**-12 months**	**0 months**	**+1 months**	**+2 months**	**>3 months**	**+27 months**	**+29 months**	**+34 months**
**RHIVA2 trial:**			X							
**Allocation**				X						
**Intervention**					X					
**Enrollment**						X	X	X	X	
**RHIVA2 post-trial implementation:**		X								
**Allocation**				X						
**Intervention**					X					
**Enrollment**						X	X	X	X	X
**Tower Hamlets:**	X									
**Allocation**				X						
**Intervention**				X						
**Enrollment**				X	X	X	X	X		
**Assessments:**										
**HIV testing***	X	X	X	X	X	X	X	X	X	X
**HIV diagnosis***	X	X	X	X	X	X	X	X	X	X

For peer review only

STANDARD PROTOCOL ITEMS: RECOMMENDATIONS FOR INTERVENTIONAL TRIALS

SPIRIT 2013 Checklist: Recommended items to address in a clinical trial protocol and related documents*

Section/item	Item No	Description	Addressed on page number
**Administrative information**			
Title	1	Descriptive title identifying the study design, population, interventions, and, if applicable, trial acronym	1
Trial registration	2a	Trial identifier and registry name. If not yet registered, name of intended registry	12
	2b	All items from the World Health Organization Trial Registration Data Set	NA
Protocol version	3	Date and version identifier	2
Funding	4	Sources and types of financial, material, and other support	15
Roles and responsibilities	5a	Names, affiliations, and roles of protocol contributors	1,2
	5b	Name and contact information for the trial sponsor	2
	5c	Role of study sponsor and funders, if any, in study design; collection, management, analysis, and interpretation of data; writing of the report; and the decision to submit the report for publication, including whether they will have ultimate authority over any of these activities	16
	5d	Composition, roles, and responsibilities of the coordinating centre, steering committee, endpoint adjudication committee, data management team, and other individuals or groups overseeing the trial, if applicable (see Item 21a for data monitoring committee)	16

For peer review only - http://bmjopen.bmj.com/site/about/guidelines.xhtml

**Introduction**			
Background and rationale	6a	Description of research question and justification for undertaking the trial, including summary of relevant studies (published and unpublished) examining benefits and harms for each intervention	4_____________
	6b	Explanation for choice of comparators	5_____________
Objectives	7	Specific objectives or hypotheses	5_____________
Trial design	8	Description of trial design including type of trial (eg, parallel group, crossover, factorial, single group), allocation ratio, and framework (eg, superiority, equivalence, noninferiority, exploratory)	5___________
**Methods: Participants, interventions, and outcomes**			
Study setting	9	Description of study settings (eg, community clinic, academic hospital) and list of countries where data will be collected. Reference to where list of study sites can be obtained	5_____________
Eligibility criteria	10	Inclusion and exclusion criteria for participants. If applicable, eligibility criteria for study centres and individuals who will perform the interventions (eg, surgeons, psychotherapists)	5_____________
Interventions	11a	Interventions for each group with sufficient detail to allow replication, including how and when they will be administered	6,7____________
	11b	Criteria for discontinuing or modifying allocated interventions for a given trial participant (eg, drug dose change in response to harms, participant request, or improving/worsening disease)	NA____________
	11c	Strategies to improve adherence to intervention protocols, and any procedures for monitoring adherence (eg, drug tablet return, laboratory tests)	NA____________
	11d	Relevant concomitant care and interventions that are permitted or prohibited during the trial	NA____________
Outcomes	12	Primary, secondary, and other outcomes, including the specific measurement variable (eg, systolic blood pressure), analysis metric (eg, change from baseline, final value, time to event), method of aggregation (eg, median, proportion), and time point for each outcome. Explanation of the clinical relevance of chosen efficacy and harm outcomes is strongly recommended	7,8____________
Participant timeline	13	Time schedule of enrolment, interventions (including any run-ins and washouts), assessments, and visits for participants. A schematic diagram is highly recommended (see Figure)	19_____________

For peer review only - http://bmjopen.bmj.com/site/about/guidelines.xhtml

Sample size	14	Estimated number of participants needed to achieve study objectives and how it was determined, including clinical and statistical assumptions supporting any sample size calculations	9_____________
Recruitment	15	Strategies for achieving adequate participant enrolment to reach target sample size	5_____________
**Methods: Assignment of interventions (for controlled trials)**			
Allocation:			
Sequence generation	16a	Method of generating the allocation sequence (eg, computer-generated random numbers), and list of any factors for stratification. To reduce predictability of a random sequence, details of any planned restriction (eg, blocking) should be provided in a separate document that is unavailable to those who enrol participants or assign interventions	Leber et al, 2015
Allocation concealment mechanism	16b	Mechanism of implementing the allocation sequence (eg, central telephone; sequentially numbered, opaque, sealed envelopes), describing any steps to conceal the sequence until interventions are assigned	Leber et al, 2015
Implementation	16c	Who will generate the allocation sequence, who will enrol participants, and who will assign participants to interventions	Leber et al, 2015
Blinding (masking)	17a	Who will be blinded after assignment to interventions (eg, trial participants, care providers, outcome assessors, data analysts), and how	Leber et al, 2015
	17b	If blinded, circumstances under which unblinding is permissible, and procedure for revealing a participant's allocated intervention during the trial	Leber et al, 2015
**Methods: Data collection, management, and analysis**			
Data collection methods	18a	Plans for assessment and collection of outcome, baseline, and other trial data, including any related processes to promote data quality (eg, duplicate measurements, training of assessors) and a description of study instruments (eg, questionnaires, laboratory tests) along with their reliability and validity, if known. Reference to where data collection forms can be found, if not in the protocol	8,9____________ _
	18b	Plans to promote participant retention and complete follow-up, including list of any outcome data to be collected for participants who discontinue or deviate from intervention protocols	9____________

For peer review only - http://bmjopen.bmj.com/site/about/guidelines.xhtml

Data management	19	Plans for data entry, coding, security, and storage, including any related processes to promote data quality (eg, double data entry; range checks for data values). Reference to where details of data management procedures can be found, if not in the protocol	9_____________
Statistical methods	20a	Statistical methods for analysing primary and secondary outcomes. Reference to where other details of the statistical analysis plan can be found, if not in the protocol	10, 11_________
	20b	Methods for any additional analyses (eg, subgroup and adjusted analyses)	10_____________
	20c	Definition of analysis population relating to protocol non-adherence (eg, as randomised analysis), and any statistical methods to handle missing data (eg, multiple imputation)	NA____________
**Methods: Monitoring**			
Data monitoring	21a	Composition of data monitoring committee (DMC); summary of its role and reporting structure; statement of whether it is independent from the sponsor and competing interests; and reference to where further details about its charter can be found, if not in the protocol. Alternatively, an explanation of why a DMC is not needed	NA____________
	21b	Description of any interim analyses and stopping guidelines, including who will have access to these interim results and make the final decision to terminate the trial	NA____________
Harms	22	Plans for collecting, assessing, reporting, and managing solicited and spontaneously reported adverse events and other unintended effects of trial interventions or trial conduct	NA____________
Auditing	23	Frequency and procedures for auditing trial conduct, if any, and whether the process will be independent from investigators and the sponsor	NA____________
**Ethics and dissemination**			
Research ethics approval	24	Plans for seeking research ethics committee/institutional review board (REC/IRB) approval	12_____________
Protocol amendments	25	Plans for communicating important protocol modifications (eg, changes to eligibility criteria, outcomes, analyses) to relevant parties (eg, investigators, REC/IRBs, trial participants, trial registries, journals, regulators)	12____________

For peer review only - http://bmjopen.bmj.com/site/about/guidelines.xhtml

Consent or assent	26a	Who will obtain informed consent or assent from potential trial participants or authorised surrogates, and how (see Item 32)	Leber et al, 2015
	26b	Additional consent provisions for collection and use of participant data and biological specimens in ancillary studies, if applicable	NA_____________
Confidentiality	27	How personal information about potential and enrolled participants will be collected, shared, and maintained in order to protect confidentiality before, during, and after the trial	12_____________
Declaration of interests	28	Financial and other competing interests for principal investigators for the overall trial and each study site	15_____________
Access to data	29	Statement of who will have access to the final trial dataset, and disclosure of contractual agreements that limit such access for investigators	16_____________
Ancillary and post-trial care	30	Provisions, if any, for ancillary and post-trial care, and for compensation to those who suffer harm from trial participation	NA_____________
Dissemination policy	31a	Plans for investigators and sponsor to communicate trial results to participants, healthcare professionals, the public, and other relevant groups (eg, via publication, reporting in results databases, or other data sharing arrangements), including any publication restrictions	3_____________
	31b	Authorship eligibility guidelines and any intended use of professional writers	15_____________
	31c	Plans, if any, for granting public access to the full protocol, participant-level dataset, and statistical code	16_____________
**Appendices**			
Informed consent materials	32	Model consent form and other related documentation given to participants and authorised surrogates	NA_____________
Biological specimens	33	Plans for collection, laboratory evaluation, and storage of biological specimens for genetic or molecular analysis in the current trial and for future use in ancillary studies, if applicable	NA_____________

*It is strongly recommended that this checklist be read in conjunction with the SPIRIT 2013 Explanation & Elaboration for important clarification on the items. Amendments to the protocol should be tracked and dated. The SPIRIT checklist is copyrighted by the SPIRIT Group under the Creative Commons "Attribution-NonCommercial-NoDerivs 3.0 Unported" license.

For peer review only - http://bmjopen.bmj.com/site/about/guidelines.xhtml

BMJ Open

Effectiveness and cost effectiveness of implementing HIV testing in primary care in east London: protocol for an interrupted time series analysis

Journal:	*BMJ Open*
Manuscript ID	bmjopen-2017-018163.R1
Article Type:	Protocol
Date Submitted by the Author:	08-Sep-2017
Complete List of Authors:	Leber, Werner; Queen Mary University of London, Centre for Primary Care and Public Health<br>Beresford, Lee; Queen Mary University of London<br>Nightingale, C; St George's University London, Division of Population Health Sciences and Education<br>Capelas Barbosa, Estela; University College London<br>Morris, Stephen; University College London, Department of Applied Health Research<br>El-Shogri, Farah; Queen Mary University of London<br>McMullen, Heather; Queen Mary University of London, Blizard Institute<br>Boomla, Kambiz; Barts and The London School of Medicine and Dentistry, Queen Mary University of London, Centre for Primary Care and Public Health<br>Delpech, Valerie; Public Health England<br>Brown, Alison; Public Health England<br>Hutchinson, Jane; Barts Health NHS Trust<br>Apea, Vanessa; Barts Health NHS Trust<br>Symonds, Merle; Barts Health NHS Trust<br>Gilliham, Smantha; Barts Health NHS Trust<br>Creighton, S.; Homerton Univ Hosp<br>Shahmanesh, Maryan; University College London<br>Fulop, Naomi; University College London, Applied Health Research<br>Estcourt, Claudia; Glasgow Caledonian University; Barts Health NHS Trust<br>Anderson, Jane ; Homerton University Hospital, Centre for the study of Sexual Health and HIV<br>Figueroa, Jose; NHS England<br>Griffiths, Chris; Barts and The London School of Medicine and Dentistry,
**Primary Subject Heading**:	General practice / Family practice
Secondary Subject Heading:	HIV/AIDS, Public health
Keywords:	PRIMARY CARE, HIV & AIDS < INFECTIOUS DISEASES, Public health < INFECTIOUS DISEASES

For peer review only

Effectiveness and cost effectiveness of implementing HIV testing in primary care in east London: protocol for an interrupted time series analysis

Werner Leber[1]

Lee Beresford[1]

Claire Nightingale[2]

Estela Capelas Barbosa[3]

Stephen Morris[3]

Farah El-Shogri[1]

Heather McMullen[1]

Kambiz Boomla[1]

Valerie Delpech[4]

Alison Brown[4]

Jane Hutchinson[5]

Vanessa Apea[5]

Merle Symonds[5]

Samantha Gilliham[5]

Sarah Creighton[6]

Maryam Shahmanesh[3]

Naomi Fulop[3]

Claudia Estcourt[5,7]

Jane Anderson[6]

Jose Figueroa[8]

Chris Griffiths[1]

1. Centre for Primary Care and Public Health, Queen Mary University of London
2. Population Health Research Institute, St George's, University of London
3. Department of Applied Health Research, University College London
4. Public Health England
5. Barts Health NHS Trust
6. Centre for Sexual Health, Homerton University Hospital NHS Foundation Trust
7. Glasgow Caledonian University

For peer review only

For peer review only - http://bmjopen.bmj.com/site/about/guidelines.xhtml

8. NHS England

Correspondence to:

Werner Leber

Centre for Primary Care and Public Health

Barts and The London School of Medicine & Dentistry

Queen Mary University of London, London E1 2AB

w.leber@qmul.ac.uk

Sponsor: Mr Gerry Leonard, Queen Mary University of London,

gerry.leonard@bartshealth.nhs.uk

Key words: HIV; HIV testing; HIV screening; HIV diagnosis; implementation; interrupted time series; cost-effectiveness

Protocol version: v5

Date: 04 Sep, 2017

Abstract

Introduction

HIV remains underdiagnosed. Guidelines recommend routine HIV testing in primary care but evidence on implementing testing is lacking. In a previous study, the RHIVA2 cluster randomised controlled trial, we showed that providing training and rapid point-of-care HIV testing at general practice registration (***RHIVA2 intervention***) in Hackney led to cost-effective, increased, and earlier diagnosis of HIV. However, interventions effective in a trial context may be less so when implemented in routine practice. We describe the protocol for an MRC phase IV implementation programme, evaluating the impact of rolling out the ***RHIVA2 intervention*** in a post-trial setting. We will use a longitudinal study to examine if the post-trial implementation in Hackney practices is effective and cost-effective; and a cross-sectional study to compare Hackney to two adjacent boroughs providing usual primary care (Newham) and an enhanced service promoting HIV testing in primary care (Tower Hamlets).

Methods and analysis

For peer review only - http://bmjopen.bmj.com/site/about/guidelines.xhtml

Service evaluation using interrupted time series and cost-effectiveness analyses. We will include all general practices in three contiguous high HIV prevalence east London boroughs. All adults aged 16 and above registered with the practices will be included. The interventions to be examined are: a post-trial RHIVA2 implementation programme (including practice-based education and training, external quality assurance, incentive payments for rapid HIV testing, and incorporation of rapid HIV testing in the sexual health Local Enhanced Service) in Hackney; the general practice sexual health Network Improved Service in Tower Hamlets; and usual care in Newham. Co-primary outcomes are rates of HIV testing and new HIV diagnoses.

Ethics and dissemination

The chair of the Camden & Islington NHS Research Ethics Committee, London, has endorsed this programme as an evaluation of routine care. Study results will be published in peer-reviewed journals and reported to commissioners.

Strengths and limitations of this study:

- The study will use existing trial data preceding the implementation programme in addition to post-trial data, collected using similar methods to those used in the trial.
- Fully anonymised general practice data from all participating boroughs will be remotely extracted using a single general practice computer system (Egton Medical Information Systems, EMIS).
- A large dataset with at least 12 time points before and at least 27 time points after the implementation will provide precise estimation of the effects of intervention using interrupted time series analysis.
- Differences in HIV testing protocols, data quality and extraction between Hackney, and Tower Hamlets and Newham; and previous interventions such as rapid HIV testing pilots in a small number of Newham practices, may affect comparability between boroughs. Also, cross-contamination of RHIVA2 control practices from intervention practices, or between boroughs, may have occurred.
- Finally, the post-trial implementation programme of the RHIVA2 intervention differed slightly from the research intervention to account for the complexities of delivering services in routine health care settings.

For peer review only

Introduction

HIV prevalence is increasing, with over 100,000 people now estimated to be living with HIV in the UK.[1] From 1999 to 2013, the numbers of patients accessing specialist care increased four-fold.[2 3] Successfully treated, HIV now has characteristics of a long-term condition, with patients taking lifelong treatment and increasingly exhibiting age related co-morbidities such as cardiovascular disease, osteoporosis and mental illness. The cost of HIV care for the NHS (without considering the cost of treating co-morbidities) in 2013 was estimated to be £750m (£1bn with social care included).[4]

Late diagnosis of HIV increases morbidity and mortality, and is associated with unwitting onward transmission. To reduce the pool of undiagnosed infection in the population, the British HIV Association (BHIVA, 2008) and the National Centre for Health and Care Excellence (NICE, 2011, 2016) recommended expansion of routine HIV testing from sexual health and antenatal clinics to non-traditional settings including general practices located in high HIV prevalence areas (two or more people diagnosed with HIV/1000 adult population).[5-8] In the Framework for Sexual Health Improvement in England (2013), the UK Department of Health additionally set out recommendations for the commissioning of HIV testing in high prevalence areas across all health care settings, including general practice.[9] However, the decision to implement these recommendations lies with the 74 local authorities in England and the respective clinical commissioning groups (CCGs). Although National Policy for HIV screening in antenatal settings has proven highly effective, guidance for screening in general practice is lacking. Indeed, it was hoped that recommending routine HIV testing would suffice to enhance awareness of HIV amongst general practitioners (GPs) and become standard in this setting.[10] Pilot studies have demonstrated feasibility and acceptability of routine HIV testing amongst patients and health care professionals.[11 12] Despite this, a recent systematic review identified low uptake of routine HIV testing in non-traditional UK settings including general practice following the BHIVA 2008 guidelines.[13]

In a large-scale pragmatic cluster randomised controlled trial (RHIVA2) in the London borough of Hackney, we showed that rapid HIV testing offered at general practice registration was effective and safe, and delivered increased and earlier HIV diagnosis.[14] Furthermore, a recent health economics analysis of the RHIVA2 trial demonstrated that HIV screening in primary care in high HIV prevalence areas is cost-effective.[15] Following

For peer review only - http://bmjopen.bmj.com/site/about/guidelines.xhtml

completion of this trial, we implemented HIV testing into routine care across all Hackney practices using a theory-based programme[16-18] comprising an adaptation of the RHIVA2 intervention, including a practice-based education programme, a borough-wide audit of HIV care, and the introduction of payments for routine HIV testing as part of an updated general practice sexual health Local Enhanced Service (LES).[19]

We will evaluate both the RHIVA2 trial and post-trial implementation programme through an MRC phase IV implementation study.[20] The study will also compare the impact of RHIVA2 implementation in Hackney practices with two neighbouring boroughs, Tower Hamlets that offers a general practice sexual health Network Improved Service (NIS) promoting routine HIV testing and Newham, offering usual primary care without incentivised HIV testing.

Figure 1 summarises the timelines of HIV-related interventions in these three boroughs.

The study will therefore address the following questions:

- How effective and cost-effective is the RHIVA2 intervention when implemented as a clinical service outside a research context?
- How do effectiveness and cost-effectiveness of the RHIVA2 trial and implementation programme compare with a borough-wide sexual health NIS promoting routine HIV testing in Tower Hamlets?
- How do both the RHIVA2 trial and post-trial implementation programme and the Tower Hamlets sexual health NIS compare to Newham that offers usual HIV primary care only?

Methods and analysis

Setting

The study will be set in the three east London boroughs of Hackney, Tower Hamlets, and Newham. The estimated diagnosed HIV prevalence for these boroughs is 8.1, 6.5, and 6.7 per 1000 adult population respectively.[21] We will include all adults aged 16 and above registered with general practices in the three boroughs.

For peer review only - http://bmjopen.bmj.com/site/about/guidelines.xhtml

Design

Service evaluation comprising a pragmatic cohort using an interrupted time series (ITS) analysis and cost effectiveness analysis. The evaluation will examine the longitudinal impact of interventions in Hackney and the comparative cross-sectional impact of interventions in Hackney, Tower Hamlets, and Newham. HIV testing and diagnosis data from 01 April, 2009 to 30 June, 2015 is being collected.

Interventions

Hackney

The RHIVA2 intervention was first introduced in April 2010 in 20 general practices randomized to the intervention arm of the RHIVA2 trial (Table 1). This theory-based intervention[16-18] is described in detail in Leber et al. 2015,[14] and consisted of a practice-based educational training session for the primary care team to promote rapid HIV testing at registration, a follow up meeting with a nominated practice lead nurse, integration of rapid HIV testing with the general practice computer template, and an incentive payment of £10 per rapid HIV test performed. Testing competence was monitored by an external quality assessment. This intervention was offered in addition to an existing national antenatal HIV screening service offered at the practices, and a sexual health LES promoting sexual health screening including HIV case detection (incentive payments of £265 per newly diagnosed patient) introduced in 2006/07.[19]

The RHIVA2 post-trial implementation programme ran from September 2012 to April 2015 and comprised the following three components:[16-18]

1) Additional post-trial HIV testing training (September 2012 to February 2013). This was offered to all Hackney practices at the completion of the RHIVA2 trial and comprised a modification of the RHIVA2 trial intervention whereby practices were encouraged to offer both routine serology and rapid HIV testing in any clinical setting (rather than just rapid testing at practice registration).
2) Updated sexual health LES. From April 2013, the existing LES was replaced with HIV testing payments to practices of between £7 and £10 for every rapid or serology

For peer review only - http://bmjopen.bmj.com/site/about/guidelines.xhtml

For peer review only

HIV test carried out in any clinical setting, in addition to a payment of £258 for any new diagnosis.

3) General practice based HIV care audit (October 2012 to March 2013) of missed diagnoses of HIV and safety of co-prescribing with anti-retroviral medication in Hackney general practices.[22]

Tower Hamlets

In April 2011, Tower Hamlets implemented sexual health NIS incentive payments promoting cooperation between practices in 8 practice networks, comprising 4-5 practices each.
The NIS insentivised sexually transmitted infections (STI) testing with separate payments for serology (£10 per HIV test combined with or without Hepatitis B and syphilis testing) and swabs/urine (£15 for Gonorrhoea and Chlamydia testing).
Practices were encouraged to offer HIV & STI testing as part of the New Patient Check as well as in sexual health & contraception consultations and opportunistically in general consultations.

In contrast to Hackney, where practices received the implementation individually over a six-month period, the Tower Hamlets sexual health NIS went live through activation of the sexual health template on the same day for all practices (April 2011). A primary care facilitator in sexual health (JH since September 2012) has additionally provided regular support, training and performance feedback to the practice networks.

Newham

Usual care – no specific initiatives were promoted relating to HIV testing in primary care.

The RHIVA2 intervention is based on published clinician behaviour change programmes,[16-18] and the Tower Hamlets NIS follows Royal College of General Practitioners guidance on establishing general practice federations.[23] We will report our study in accordance with the STARI reporting guidelines for implementation studies.[24]

Outcome measures

For peer review only - http://bmjopen.bmj.com/site/about/guidelines.xhtml

All data gathered will comprise routine clinical data. No identifiable data will be held by the research team.

Co-primary outcome measures

Co-primary outcome measures will be:

- Rates of HIV testing (combined rapid and serology testing; number of patients tested/1000 population per year)
- Rates of new HIV diagnosis (number of newly diagnosed patients/10,000/year)

Secondary outcome measures

Secondary outcome measures will include data on type of HIV test (serology; rapid), location of diagnosis, stage of disease and linkage to secondary care and retention in secondary care:

- Number / rate of patients who received a rapid HIV test
- Number / rate of patients who received a serology HIV test
- Number / rate of patients newly diagnosed in general practice
- Mean and median CD4 count of patients newly diagnosed in primary care
- Proportion of patients with a CD4 count <350 cells per µL blood
- Proportion of patients with a CD4 count <200 cells per µL blood.
- The proportion of patients newly diagnosed with HIV in primary care that attend an HIV specialist department within 1 week of diagnosis.
- The proportion of patients newly diagnosed with HIV in primary care that attend an HIV specialist department within 2 weeks of diagnosis (BHIVA Standards of Care for PLHIV, Standard 2, 2013).[25]
- The proportion of patients newly diagnosed with HIV in primary care that attend an HIV specialist department within 4 weeks of diagnosis (NHS England Standard)
- The proportion of patients newly diagnosed with HIV in primary care that attend a HIV specialist department within 3 months of diagnosis (Optimising testing and linkage to care for HIV across Europe, OptTEST, 2013)
- Number of patients previously known to have HIV, who attend an HIV specialist clinic between 12 and 24 months following new diagnosis in primary care (BHIVA Standards of Care for PLHIV, Standard 2, 2013).

For peer review only - http://bmjopen.bmj.com/site/about/guidelines.xhtml

Data sources

We will gather data from April 2009 to June 2015 using the following sources:

Organisational data:

For Hackney practices, we hold detailed records of RHIVA2 trial educational training sessions, post-trial education sessions, and participation in the borough-wide audit of HIV care. For Tower Hamlets, we hold data on timing of the introduction of the NIS. For all boroughs, we will seek to obtain any other relevant activities promoting HIV testing in the practices.

Clinical data:

HIV diagnosis data

Borough-specific master case-report forms (CRF) containing lists of patients with a positive confirmatory HIV test result will be generated by the lead HIV clinician from the clinical HIV services in each borough. This master CRF will be shared with the study team in an anonymised format as per the RHIVA2 protocol. At Homerton Hospital, Hackney data on newly diagnosed patients will be collected prospectively as outlined in RHIVA2 (continuous data available from 2009). For Tower Hamlets and Newham, new diagnosis data will be collected retrospectively for the same period. Clinical leads will liaise with Public Health England to categorise patients with a positive confirmatory HIV test into newly diagnosed patients and people already known to be HIV positive, and obtain data on patients who had defaulted from care between 12 and 24 months following diagnosis.

HIV testing data

In collaboration with the Clinical Effectiveness Group in the Centre for Primary Care and Public Health (QMUL), we will collect anonymised data on HIV testing in primary care, as per RHIVA2 protocol, using remote searches of borough-specific READ codes used for rapid and serology HIV testing.

Sample size and analysis

For peer review only - http://bmjopen.bmj.com/site/about/guidelines.xhtml

Sample size

Sample size calculations for the interrupted time series (ITS) analysis are based on the number of time points available to perform the analysis. Generally, a minimum of 10-12 time points before and after an intervention are required to determine statistical significance of an intervention.[26 27] 12 monthly time points are available before the RHIVA2 intervention, and a minimum of 34 time points for the period thereafter. For Tower Hamlets, 21 monthly time points are available before the introduction of the sexual health NIS, a minimum of 27 time points thereafter.

Longitudinal analysis of effectiveness of RHIVA2 implementation programme in Hackney

We will compare testing rates using an interrupted time series regression approach with a mixed Poisson model (or negative binomial model if there is overdispersion) to analyse the effect of interventions. Mixed Poisson regression will allow for the clustered nature of the data, i.e. allowing each practice to have a different intercept (baseline HIV testing rate). We will also adjust for practice list size as an offset variable in the analysis. Using this approach, it will be possible to examine whether there is a change in level and/or a change in regression slope following the implementation of the intervention.

We will firstly examine the impact of the RHIVA2 intervention on combined serology and rapid HIV testing rate in practices that received the RHIVA2 implementation (post-trial setting, comparing testing rates pre- and post-intervention). We will repeat this analysis including practices which did not receive the RHIVA2 intervention during the trial or the further implementation phase, to act as a comparator. This will allow comparison of testing rates in practices post-implementation to their pre-implementation rates, also adjusting for continuing trends in practices that did not receive the RHIVA2 implementation. We will also repeat the analysis using Newham practices (which received no intervention) as a further comparator.

The date of intervention will be defined as the date of the first RHIVA2 training session. We will conduct similar analysis to the above, with the endpoint of HIV diagnosis, rather than testing.

For peer review only - http://bmjopen.bmj.com/site/about/guidelines.xhtml

To assess sustainability, we will model testing rates in practices that received the RHIVA2 intervention during the trial period, considering an interaction term between time post-intervention and whether practices were retrained. This will allow an assessment to be made about whether retraining influences the slope post-intervention i.e. the sustainability of the RHIVA2 intervention.

Cross-sectional analysis of effectiveness of the RHIVA2 implementation programme in Hackney, a sexual health NIS in Tower Hamlets, and usual care in Newham

We will examine the impact of the sexual health NIS on the serology testing rate in all Tower Hamlets practices (comparing testing rates pre- and post-implementation). We will repeat this analysis but also including practices in Newham, which received no intervention at any stage, to act as a comparator. The date of intervention will be defined as the date when the sexual health NIS system went live for all practices (April 2011). We will also compare the RHIVA2 implementation in Hackney to the introduction of the sexual health NIS in Tower Hamlets.

We will conduct similar analysis to the above, with the endpoint of HIV diagnosis, rather than testing.

Cost-effectiveness analysis

Cost-effectiveness analyses for the longitudinal and cross-sectional analyses described above will build on a cost-effectiveness model developed for the RHIVA2 trial.[15] To assess value for money, we will identify the difference in costs and the difference in benefits of implementing the RHIVA2 intervention and we aim to distinguish between two different analyses. First, we will evaluate the cost-effectiveness of the RHIVA2 implementation using the same ‘comparator’ defined in the longitudinal analysis, i.e. practices which did not receive the RHIVA2 intervention at any stage will be considered the ‘absence of the intervention’.

Second, we will build a cost-effectiveness comparison between different forms of HIV screening in east London, formally comparing the RHIVA2 implementation (conducted in

For peer review only - http://bmjopen.bmj.com/site/about/guidelines.xhtml

Hackney) with the sexual health NIS (Tower Hamlets) and usual care (Newham). Once again, all three different approaches will be compared to practices, which did not receive the RHIVA2 intervention at any stage, as these are considered the 'absence of the intervention'. Given that the same control group of practices in Hackney will serve as reference, this second analysis will formally compare the different approaches from different boroughs, considering no intervention in Hackney as a reference.

Three main cost categories will be explored: (1) the extra start-up cost of RHIVA2 implementation (specifically the cost for RHIVA2 staff time, including follow up activities; cost of training; cost of training materials; and cost of incentives to practices); (2) the extra recurrent cost for performing rapid HIV testing; and (3) additional costs associated with reactive rapid HIV test results.

Benefits of the RHIVA2 programme will include health outcomes for patients, specifically deaths averted, secondary HIV infections averted and quality adjusted life years (QALYs); these are a function of the uptake of screening and screening positivity, which will be quantified directly by the analyses.

This study is a service evaluation which does not require registration as a trial.

Ethical aspects

The programme described is an evaluation of routine care. Its status as such has been endorsed by the chair of the Camden & Islington NHS Research Ethics Committee London. The Committee's view is that the work does not require NHS ethics approval. The programme will be implemented in partnership with the local providers and commissioners and involve the analysis of existing routine data. This also applies to data on delayed diagnosis of HIV (CD4 count), and demographics reported in RHIVA2 trial, which is routinely collected as part of a national audit (BHIVA National Audit 2010) and has been included in Public Health Outcomes Framework (http://www.phoutcomes.info). We will inform the REC, the CCG, and Public Health about any important changes to the protocol.

For peer review only - http://bmjopen.bmj.com/site/about/guidelines.xhtml

This service evaluation is to answer the question: what standard does this service achieve? It involves routine implementation of an intervention outside of the research context. The actual management options are those of the clinician and patient only. There is no random allocation to intervention and control groups. No individual consent is required from newly diagnosed patients.

In addition to CD4 count and viral load the following additional data will be obtained by the clinical leads of specialist departments in an anonymized fashion and stored in accordance with good clinical practice:

- Most likely mode of HIV acquisition
- Sexual orientation
- Soundex code
- Ethnicity
- Gender
- Age at diagnosis

Discussion

The MRC Framework for Complex Interventions[28] clearly describes the need for implementation studies that test the effectiveness of interventions found to be promising in Phase III randomized controlled trials. Successful translation of such interventions into routine care cannot be assumed; trials take place in tightly controlled conditions that aim to limit bias, with selected (often motivated) participants, whilst implementation programmes (usually) aim much more broadly to include all potential participants.[29] Trial interventions will often need to be augmented by a distinct implementation package. Their success will be affected by multiple factors including, but not limited to contextual factors such as, differences in setting, population, organization of health care systems, competing initiatives, and financial aspects (including availability of incentives). Evaluations of implementation of trials into routine care are frequently not carried out, and if they are, are often poorly reported.[30] Such evaluations are highly unlikely to involve randomised designs, and are more likely to be observational, including the use of service evaluations. Designs are often constrained by health systems and data availability, but need to be as robust as possible. New reporting guidelines have recently been published that highlight the need for evaluation of

For peer review only - http://bmjopen.bmj.com/site/about/guidelines.xhtml

implementation and aim to improve their reporting, such that they can be more easily accessed in the literature and serve to guide others in designing the most effective implementation programmes.[29]

Recent NICE Guidelines recommend expanded HIV testing in primary care,[6-8] but little information is available about how to operationalize testing, especially in the context of current resource constraints in primary care. It is therefore vital that innovative and effective ways are found to implement testing. The strengths of the proposed work include the availability of existing trial data preceding the implementation programme, the capacity to collect data longitudinally using similar methods to those used in the trial, and the use by general practices across all three participating boroughs of a single GP computer system supplier (EMIS), data from which can be remotely and electronically (with full anonymisation) searched and collated by the research team (http://www.blizard.qmul.ac.uk/ceg-home.html), confirmation of HIV test results for all study practices at the same tertiary care laboratory (Barts Health NHS Trust), and external data validation by Public Health England. This approach provides a unique capability, allowing comparative data on three separate approaches to HIV testing and diagnosis to be accurately gathered and analysed.

Due to difference in data extraction between Hackney, and Tower Hamlets and Newham respectively, there may be a small chance of differential reporting bias between these boroughs. In contrast to the Department of Sexual Health at Homerton University Hospital (Hackney), which is able to track any patient lost to follow using a monthly list of HIV positives provided by the lab, no such failsafe procedure exists in Tower Hamlets or Newham. However, in these latter boroughs all data for HIV positive test results are available on the general practice computer system via an electronic lab-link, almost eliminating the chance of missing data. We are currently conducting an audit of outcomes from people newly diagnosed with HIV in Tower Hamlets and Newham general practices to extract this data for the study period specified.

We cannot exclude occurrence of any cluster or cross-over effects, although we would expect for this to be minimal given that we detected a more than four-fold increase in HIV testing rates between the RHIVA2 trial intervention and control practices. In contrast to the network

For peer review only - http://bmjopen.bmj.com/site/about/guidelines.xhtml

structure in Tower Hamlets, Hackney practices, which were randomly allocated to the trial arms, may have informally worked collaboratively through geographical proximity. Due to this potential clustering effect, inference of generalisability of RHIVA2 findings in other boroughs should therefore be treated with caution. Furthermore, an audit of new diagnosis and safe co-prescribing was also conducted by our team across 31 of the 44 Hackney practices during the implementation phase¸ potentially causing contamination of control practices. However, the number of both trial intervention (N=13) and control (N=15) practices was comparable, suggesting equal exposure to this audit across both arms.[22] Although public health teams informally collaborate across northeast London, cross-over between boroughs is unlikely given that services are developed and budgeted separately. Our analysis will adjust for any potential contamination using various vertical and horizontal controls both within Hackney, as well as between boroughs using Newham, which has been devoid of any local service HIV promotional intervention, as control.

Moreover, the RHIVA2 trial and post-trial implementation will not be grouped as the same intervention. We will only use the post-trial implementation practices in the main analysis and only consider the trial intervention practices for the assessment of sustainability. Therefore, the sustainability analysis will have to be interpreted with caution with regards to the sustainability of the post-trial intervention, as the intervention in trial conditions might be different to that outside of trial conditions.

We may not be able to account for missing data such as from patients lost to follow up after diagnosis in general practice, or from rapid testing pilots conducted in a small number of Newham practices during the study period.

Finally, the RHIVA2 trial intervention was adapted for the implementation phase 1) to expand promotion of routine offer of HIV testing from new registration checks to include sexual health and contraception appointments and 2) to enhance screening through additional use of venous blood sampling. This protocol adaptation was made to meet the needs of the local population through increasing access to testing, reflecting the complexities of translating a research intervention into clinical practice after completion of the research.

For peer review only - http://bmjopen.bmj.com/site/about/guidelines.xhtml

The results of the proposed analysis are likely to be of considerable interest to commissioners, particularly given that late diagnosis of HIV is a UK Public Health Outcomes Framework priority,[31] and that cost effectiveness of HIV testing is complex, and becoming cost-effective in trial-based models only after a period of time has elapsed to counter the additional significant costs of early treatment.

Study status

We have completed collection of HIV testing data for all three boroughs. The HIV diagnosis and cost-effectiveness data for this study are expected to become available in the next six to nine months.

Abbreviations

BHIVA, British HIV Association
CLAHRC, Collaboration for Leadership in Applied Health Research and Care
EMIS, Egton Medical Information Systems
HIV, Human Immunodeficiency Virus
MRC, Medical Research Council
NICE, The National Institute for Health and Care Excellence
NIHR, National Institute for Health Research
LES, Local Enhanced Service
NIS, Network Improved Service
RCT, Randomised Controlled Trial
RHIVA2, Rapid HIV Testing Assessment 2

Competing interests

JA reports fees and non-financial support from Bristol Myers Squibb, grants and personal fees from Gilead Sciences, personal fees from ViiV, personal fees from Merck Sharp & Dohme, grants from Janssen, and personal fees from AbbVie, outside the submitted work. CE

For peer review only - http://bmjopen.bmj.com/site/about/guidelines.xhtml

For peer review only

and JH report grants from Gilead Sciences. The remaining authors and contributors declare no competing interest.

Author's contributions

WL, LB, CN, ECB, SM, CE, JA, JF, and CG significantly contributed to designing the study and drafted the protocol. FES, HM, KB, VD, AB, JH, AV, MS, SG, SC, MS, and NF contributed to designing the study. All authors and contributors approved the submitted version of the manuscript.

Funding

WL, FS, LB, CN, ECB were supported by the National Institute for Health Research (NIHR) Collaboration for Leadership in Applied Health Research and Care (CLAHRC) North Thames at Barts Health NHS Trust. HM was supported by an NIHR Doctoral Fellowship from 2013 to 2016. The views expressed are those of the author(s) and not necessarily those of the NHS, the NIHR or the Department of Health. HM was supported by a National Institute of Health Research Doctoral Fellowship from 2013 to 2016.

Role of the funding source

JF, a clinician employed by NHS City and Hackney, which funded the study, was involved in designing the study, data interpretation, and writing the report, but has no role in data collection or analysis. Neither the Department of Health nor the study sponsor had any role in any aspect of the study. The corresponding author will have full access to all the data in the study and has final responsibility for the decision to submit for publication.

Roles and responsibilities

CG and WL are the co-chief investigators of the study and formed the steering committee with SM, CE, JH, MS, JA, and NF. WL is responsible for the data management. CN and LB

For peer review only - http://bmjopen.bmj.com/site/about/guidelines.xhtml

have produced the power calculation and statistical model. ECB and SM have developed the protocol for the health economics analysis.

Data Sharing Statement

This study is an evaluation of service, and no ethical approval is available for data sharing.

For peer review only

For peer review only - http://bmjopen.bmj.com/site/about/guidelines.xhtml

Figure Legends

Figure 1 Sexual health service provision in east London general practice. In Hackney (Blue), the Rapid HIV Assessment (RHIVA) research programme promoting rapid point-of-care HIV testing was developed in addition to a sexual health Local Enhanced Service (LES) and included: a pilot study (RHIVA1), a cluster randomised controlled trial across 20 intervention practices (RHIVA2), and implementation of the RHIVA2 intervention across all Hackney practices. In April 2013, the RHIVA2 intervention was integrated with the sexual health LES. In Tower Hamlets (Red), a sexual health Network Improved Service (NIS) replaced the previous LES in April 2011. Newham (Green) does not provide a service promoting HIV testing (usual care).

For peer review only

For peer review only - http://bmjopen.bmj.com/site/about/guidelines.xhtml

Table 1: Schedule for enrolment, interventions, and assessment of the study.

	STUDY PERIOD									
	Control period			Practice Allocation	Post-allocation			Close-out		
TIME POINT	-48 months	-41 months	-12 months	0 months	+1 months	+2 months	>3 months	+27 months	+29 months	+34 months
RHIVA2 trial:			X							
Allocation				X						
Intervention					X					
Enrollment						X	X	X	X	
RHIVA2 post-trial implementation:		X								
Allocation				X						
Intervention					X					
Enrollment						X	X	X	X	X
Tower Hamlets:	X									
Allocation				X						
Intervention				X						
Enrollment				X	X	X	X	X		
Assessments:										
HIV testing*	X	X	X	X	X	X	X	X	X	X
HIV diagnosis*	X	X	X	X	X	X	X	X	X	X

For peer review only

References

1. Public Health England. HIV in the UK: 2016 report, 2016.
2. Health Protection Agency. HIV in the United Kingdom: 2009 Report. 2009
3. Public Health England. HIV in the United Kingdom: 2014 report 2014 [Available from: https://www.gov.uk/government/statistics/hiv-in-the-united-kingdom accessed July 12 2015.
4. Mandalia S, Mandalia R, Lo G, et al. Rising population cost for treating people living with HIV in the UK, 1997-2013.: Public Library of Science, 2010:e15677.
5. British HIV Association. UK National Guidelines for HIV testing. 2008
6. National Institute for Health and Care Excellence. Increasing the uptake of HIV testing to reduce undiagnosed infection and prevent transmission among men who have sex with men, 2011.
7. National Institute for Health and Care Excellence. Increasing the uptake of HIV testing to reduce undiagnosed infection and prevent transmission among black African communities living in England., 2011.
8. National institute for Health and Care Excellence. HIV testing: increasing uptake among people who may have undiagnosed HIV (Joint NICE and Public Health England guideline), 2016.
9. UK Department of Health. A Framework for Sexual Health Improvement in England, 2013.
10. Kall MM, Smith RD, Delpech VC. Late HIV diagnosis in Europe: a call for increased testing and awareness among general practitioners. *Eur J Gen Pract* 2012;18(3):181-6. doi: 10.3109/13814788.2012.685069
11. Health Protection Agency. Time to Test for HIV: Expanding HIV testing in healthcare and community services in England. 2011
12. Prost A, Elford J, Imrie J, et al. Social, behavioural, and intervention research among people of Sub-Saharan African origin living with HIV in the UK and Europe: literature review and recommendations for intervention. *AIDS and behavior* 2008;12(2):170-94. doi: 10.1007/s10461-007-9237-4
13. Elmahdi R, Gerver SM, Gomez Guillen G, et al. Low levels of HIV test coverage in clinical settings in the U.K.: a systematic review of adherence to 2008 guidelines. *Sex Transm Infect* 2014;90(2):119-24. doi: 10.1136/sextrans-2013-051312
14. Leber W, McMullen H, Anderson J, et al. Promotion of rapid testing for HIV in primary care (RHIVA2): a cluster-randomised controlled trial. *The Lancet HIV* 2015;2(6):e229-e35. doi: 10.1016/S2352-3018(15)00059-4
15. Baggaley RF, Irvine MA, Leber W, et al. Cost-effectiveness of screening for HIV in primary care: a health economics modelling analysis. *Lancet HIV* 2017 doi: 10.1016/S2352-3018(17)30123-6
16. Forsetlund L, Bjorndal A, Rashidian A, et al. Continuing education meetings and workshops: effects on professional practice and health care outcomes. *The Cochrane database of systematic reviews* 2009(2):CD003030. doi: 10.1002/14651858.CD003030.pub2
17. Griffiths C, Sturdy P, Brewin P, et al. Educational outreach to promote screening for tuberculosis in primary care: a cluster randomised controlled trial. *Lancet* 2007;369(9572):1528-34. doi: S0140-6736(07)60707-7 [pii]
10.1016/S0140-6736(07)60707-7 [doi] [published Online First: 2007/05/08]

For peer review only - http://bmjopen.bmj.com/site/about/guidelines.xhtml

18. O'Brien MA, Rogers S, Jamtvedt G, et al. Educational outreach visits: effects on professional practice and health care outcomes. *The Cochrane database of systematic reviews* 2007(4):CD000409. doi: 10.1002/14651858.CD000409.pub2
19. Sohal H, Creighton S, Figueroa J, et al. The impact of establishing a local-enhanced service for treating sexually transmitted infections in primary care. *Sex Transm Infect* 2008;84(3):235-7; discussion 37-8. doi: 10.1136/sti.2007.025981 [published Online First: 2008/01/15]
20. Campbell M, Fitzpatrick R, Haines A, et al. Framework for design and evaluation of complex interventions to improve health. *BMJ* 2000;321(7262):694-6.
21. Public Health England. HIV: annual data tables. Diagnosed HIV prevalence by upper and lower tier local authority.
22. Wellesley R, Whittle A, Figueroa J, et al. Does general practice deliver safe primary care to people living with HIV? A case-notes review. *Br J Gen Pract* 2015;65(639):e655-61. doi: 10.3399/bjgp15X686905 [published Online First: 2015/09/29]
23. Royal College of General Practitioners. The 2022 GP. A Vision for General Practice in the future NHS, 2014.
24. Pinnock H, Epiphaniou E, Sheikh A, et al. Developing standards for reporting implementation studies of complex interventions (StaRI): a systematic review and e-Delphi. *Implementation Science* 2015;10(1):42.
25. British HIV Association. Standards of care for people living with HIV in 2013. 2013
26. Ramsay CR, Matowe L, Grilli R, et al. Interrupted time series designs in health technology assessment: lessons from two systematic reviews of behavior change strategies. *Int J Technol Assess Health Care* 2003;19(4):613-23.
27. Wagner AK, Soumerai SB, Zhang F, et al. Segmented regression analysis of interrupted time series studies in medication use research. *J Clin Pharm Ther* 2002;27(4):299-309. [published Online First: 2002/08/14]
28. Campbell NC, Murray E, Darbyshire J, et al. Designing and evaluating complex interventions to improve health care. *BMJ* 2007;334(7591):455-9. doi: 10.1136/bmj.39108.379965.BE
29. Pinnock H, Epiphaniou E, Sheikh A, et al. Developing standards for reporting implementation studies of complex interventions (StaRI): a systematic review and e-Delphi. *Implement Sci* 2015;10(1):42. doi: 10.1186/s13012-015-0235-z
30. Newhouse R, Bobay K, Dykes PC, et al. Methodology issues in implementation science. *Medical care* 2013;51(4 Suppl 2):S32-40. doi: 10.1097/MLR.0b013e31827feeca
31. Public Health England. Public Health Outcomes Framework: February 2015 data update, 2015.

For peer review only

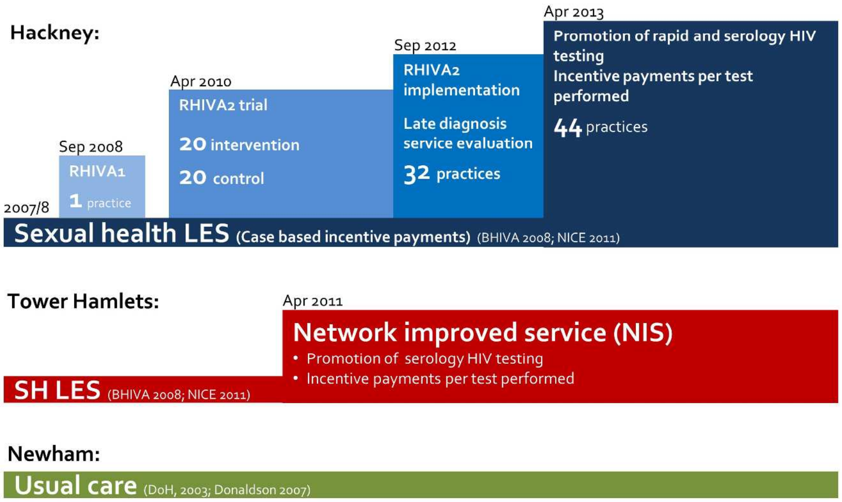

Sexual health service provision in east London general practice.

254x190mm (300 x 300 DPI)

SPIRIT

STANDARD PROTOCOL ITEMS: RECOMMENDATIONS FOR INTERVENTIONAL TRIALS

SPIRIT 2013 Checklist: Recommended items to address in a clinical trial protocol and related documents*

Section/item	Item No	Description	Addressed on page number
**Administrative information**			
Title	1	Descriptive title identifying the study design, population, interventions, and, if applicable, trial acronym	1
Trial registration	2a	Trial identifier and registry name. If not yet registered, name of intended registry	12
	2b	All items from the World Health Organization Trial Registration Data Set	NA
Protocol version	3	Date and version identifier	2
Funding	4	Sources and types of financial, material, and other support	15
Roles and responsibilities	5a	Names, affiliations, and roles of protocol contributors	1,2
	5b	Name and contact information for the trial sponsor	2
	5c	Role of study sponsor and funders, if any, in study design; collection, management, analysis, and interpretation of data; writing of the report; and the decision to submit the report for publication, including whether they will have ultimate authority over any of these activities	16
	5d	Composition, roles, and responsibilities of the coordinating centre, steering committee, endpoint adjudication committee, data management team, and other individuals or groups overseeing the trial, if applicable (see Item 21a for data monitoring committee)	16

For peer review only - http://bmjopen.bmj.com/site/about/guidelines.xhtml

Introduction

Background and rationale	6a	Description of research question and justification for undertaking the trial, including summary of relevant studies (published and unpublished) examining benefits and harms for each intervention	4_______________
	6b	Explanation for choice of comparators	5_______________
Objectives	7	Specific objectives or hypotheses	5_______________
Trial design	8	Description of trial design including type of trial (eg, parallel group, crossover, factorial, single group), allocation ratio, and framework (eg, superiority, equivalence, noninferiority, exploratory)	5____________

Methods: Participants, interventions, and outcomes

Study setting	9	Description of study settings (eg, community clinic, academic hospital) and list of countries where data will be collected. Reference to where list of study sites can be obtained	5_______________
Eligibility criteria	10	Inclusion and exclusion criteria for participants. If applicable, eligibility criteria for study centres and individuals who will perform the interventions (eg, surgeons, psychotherapists)	5_______________
Interventions	11a	Interventions for each group with sufficient detail to allow replication, including how and when they will be administered	6,7_____________
	11b	Criteria for discontinuing or modifying allocated interventions for a given trial participant (eg, drug dose change in response to harms, participant request, or improving/worsening disease)	NA_____________
	11c	Strategies to improve adherence to intervention protocols, and any procedures for monitoring adherence (eg, drug tablet return, laboratory tests)	NA_____________
	11d	Relevant concomitant care and interventions that are permitted or prohibited during the trial	NA_____________
Outcomes	12	Primary, secondary, and other outcomes, including the specific measurement variable (eg, systolic blood pressure), analysis metric (eg, change from baseline, final value, time to event), method of aggregation (eg, median, proportion), and time point for each outcome. Explanation of the clinical relevance of chosen efficacy and harm outcomes is strongly recommended	7,8_____________
Participant timeline	13	Time schedule of enrolment, interventions (including any run-ins and washouts), assessments, and visits for participants. A schematic diagram is highly recommended (see Figure)	19_____________

For peer review only - http://bmjopen.bmj.com/site/about/guidelines.xhtml

Sample size	14	Estimated number of participants needed to achieve study objectives and how it was determined, including clinical and statistical assumptions supporting any sample size calculations	9_____________
Recruitment	15	Strategies for achieving adequate participant enrolment to reach target sample size	5_____________

Methods: Assignment of interventions (for controlled trials)

Allocation:			
Sequence generation	16a	Method of generating the allocation sequence (eg, computer-generated random numbers), and list of any factors for stratification. To reduce predictability of a random sequence, details of any planned restriction (eg, blocking) should be provided in a separate document that is unavailable to those who enrol participants or assign interventions	Leber et al, 2015
Allocation concealment mechanism	16b	Mechanism of implementing the allocation sequence (eg, central telephone; sequentially numbered, opaque, sealed envelopes), describing any steps to conceal the sequence until interventions are assigned	Leber et al, 2015
Implementation	16c	Who will generate the allocation sequence, who will enrol participants, and who will assign participants to interventions	Leber et al, 2015
Blinding (masking)	17a	Who will be blinded after assignment to interventions (eg, trial participants, care providers, outcome assessors, data analysts), and how	Leber et al, 2015
	17b	If blinded, circumstances under which unblinding is permissible, and procedure for revealing a participant's allocated intervention during the trial	Leber et al, 2015

Methods: Data collection, management, and analysis

Data collection methods	18a	Plans for assessment and collection of outcome, baseline, and other trial data, including any related processes to promote data quality (eg, duplicate measurements, training of assessors) and a description of study instruments (eg, questionnaires, laboratory tests) along with their reliability and validity, if known. Reference to where data collection forms can be found, if not in the protocol	8,9_____________ _
	18b	Plans to promote participant retention and complete follow-up, including list of any outcome data to be collected for participants who discontinue or deviate from intervention protocols	9____________

Data management	19	Plans for data entry, coding, security, and storage, including any related processes to promote data quality (eg, double data entry; range checks for data values). Reference to where details of data management procedures can be found, if not in the protocol	9_____________
Statistical methods	20a	Statistical methods for analysing primary and secondary outcomes. Reference to where other details of the statistical analysis plan can be found, if not in the protocol	10, 11_________
	20b	Methods for any additional analyses (eg, subgroup and adjusted analyses)	10_____________
	20c	Definition of analysis population relating to protocol non-adherence (eg, as randomised analysis), and any statistical methods to handle missing data (eg, multiple imputation)	NA_____________
**Methods: Monitoring**			
Data monitoring	21a	Composition of data monitoring committee (DMC); summary of its role and reporting structure; statement of whether it is independent from the sponsor and competing interests; and reference to where further details about its charter can be found, if not in the protocol. Alternatively, an explanation of why a DMC is not needed	NA_____________
	21b	Description of any interim analyses and stopping guidelines, including who will have access to these interim results and make the final decision to terminate the trial	NA_____________
Harms	22	Plans for collecting, assessing, reporting, and managing solicited and spontaneously reported adverse events and other unintended effects of trial interventions or trial conduct	NA_____________
Auditing	23	Frequency and procedures for auditing trial conduct, if any, and whether the process will be independent from investigators and the sponsor	NA_____________
**Ethics and dissemination**			
Research ethics approval	24	Plans for seeking research ethics committee/institutional review board (REC/IRB) approval	12_____________
Protocol amendments	25	Plans for communicating important protocol modifications (eg, changes to eligibility criteria, outcomes, analyses) to relevant parties (eg, investigators, REC/IRBs, trial participants, trial registries, journals, regulators)	12_____________

For peer review only - http://bmjopen.bmj.com/site/about/guidelines.xhtml

Consent or assent	26a	Who will obtain informed consent or assent from potential trial participants or authorised surrogates, and how (see Item 32)	Leber et al, 2015
	26b	Additional consent provisions for collection and use of participant data and biological specimens in ancillary studies, if applicable	NA_____________
Confidentiality	27	How personal information about potential and enrolled participants will be collected, shared, and maintained in order to protect confidentiality before, during, and after the trial	12_____________
Declaration of interests	28	Financial and other competing interests for principal investigators for the overall trial and each study site	15_____________
Access to data	29	Statement of who will have access to the final trial dataset, and disclosure of contractual agreements that limit such access for investigators	16_____________
Ancillary and post-trial care	30	Provisions, if any, for ancillary and post-trial care, and for compensation to those who suffer harm from trial participation	NA_____________
Dissemination policy	31a	Plans for investigators and sponsor to communicate trial results to participants, healthcare professionals, the public, and other relevant groups (eg, via publication, reporting in results databases, or other data sharing arrangements), including any publication restrictions	3_____________
	31b	Authorship eligibility guidelines and any intended use of professional writers	15_____________
	31c	Plans, if any, for granting public access to the full protocol, participant-level dataset, and statistical code	16_____________
**Appendices**			
Informed consent materials	32	Model consent form and other related documentation given to participants and authorised surrogates	NA_____________
Biological specimens	33	Plans for collection, laboratory evaluation, and storage of biological specimens for genetic or molecular analysis in the current trial and for future use in ancillary studies, if applicable	NA_____________

*It is strongly recommended that this checklist be read in conjunction with the SPIRIT 2013 Explanation & Elaboration for important clarification on the items. Amendments to the protocol should be tracked and dated. The SPIRIT checklist is copyrighted by the SPIRIT Group under the Creative Commons "Attribution-NonCommercial-NoDerivs 3.0 Unported" license.

For peer review only - http://bmjopen.bmj.com/site/about/guidelines.xhtml
